# A novel multikinase inhibitor R8 exhibits potent inhibition on cancer cells through both apoptosis and autophagic cell death

**DOI:** 10.18632/oncotarget.20257

**Published:** 2017-08-14

**Authors:** Yuqiong Xie, Chunchun Li, Yali Huang, Zhenyu Jia, Jiang Cao

**Affiliations:** ^1^ Clinical Research Center, The Second Affiliated Hospital, Zhejiang University School of Medicine, Hangzhou 310009, China; ^2^ Institute of Hygiene, Zhejiang Academy of Medical Sciences, Hangzhou 310013, China; ^3^ Institute of Occupational Diseases, Zhejiang Academy of Medical Sciences, Hangzhou 310013, China

**Keywords:** multikinase inhibitor, targeted therapy, cancer, autophagy, apoptosis

## Abstract

Chemotherapy is an important treatment for cancer patients, especially for those with unresectable lesions. Targeted therapy of cancer by specific inhibition of aberrant tyrosine kinase activities in cancer cells with chemically synthesized tyrosine kinase inhibitors (TKIs), shows better responses while less side effects than traditional chemotherapeutic drugs. It is common that cancer cells often exhibit deregulation of several tyrosine kinases simultaneously, multikinase TKIs (MKIs) therefore have greater advantages over single-target TKIs. Currently more MKIs are under developing for better efficacy for different types of cancer. In the present work, we evaluated the *in vitro* therapeutic potential of a novel MKI, namely R8, with comparison to the clinically available MKI Sunitinib. Results showed that R8 has stronger inhibition on six different types of cancer cell lines with lower IC_50_ than Sunitinib does. Cell cycle analysis showed that R8 induced significant G0/G1 arrest phase of lung cancer A549 and NCI-H226 cells. The inhibition was also confirmed by colony formation and migration assays in both lung cancer cell lines in a dose-dependent manner. R8 could significantly inhibit the phosphorylation of multiple receptor tyrosine kinases (RTKs) included PDGFRβ, VEGFR2, EGFR and C-Kit, leading to the down-regulation of PI3K-Akt-mTOR signaling. Further analysis revealed that R8 treatment induced more significant apoptosis than Sunitinib did, which might be the consequence of the autophagic cell death. In conclusion, this work suggested R8 to be a promising novel anticancer MKI, and provided the basis for further *in vivo* investigation on its potential in treatment of lung cancer.

## INTRODUCTION

Cancer is one of the leading causes of death worldwide, with an estimated 14.1 million new cases and 8.2 million deaths occurred in 2012 worldwide [1]. Chemotherapy is an important way of treatment for cancer patients, especially for those with unresectable lesions. Targeted therapy of cancer by specific inhibition of aberrantly activated receptor tyrosine kinase (RTK) activities in cancer cells with chemically synthesized small molecule compounds, tyrosine kinase inhibitors (TKIs), shows better responses while less side effects than traditional chemotherapeutic drugs. It is common that cancer cells often exhibit simultaneous deregulation of several tyrosine kinases, such as epithelial growth factor receptor (EGFR), platelet-derived growth factor receptor (PDGFR), vascular endothelial growth factor receptor (VEGFR), *etc.*, which lead to the malignant characteristics of tumor such as uncontrolled proliferation, invasiveness and angiogenesis, multi-target TKIs (or multikinase inhibitors, MKIs) therefore have great advantages over single-target TKIs [2].

Although many TKIs/MKIs have been widely used clinically in the treatment of various types of cancer, they do not always give satisfactory outcomes, even for individuals with definite hyper-activated target kinases. Primary resistance and acquired resistance are commonly observed because of the fact that, each type of cancer or even each individual cancer cell may show different aberrantly activated tyrosine kinases due to heterogenicity during the development and progression of the disease, and the TKIs/MKIs may also have different binding and inhibition selectivities on different kinases (and even same kinase with different mutations) [3–5]. Therefore more multi-target TKIs are developed or under developing for better efficacies on different individuals and to overcome the primary or acquired resistance [6–8].

**Figure 1:**
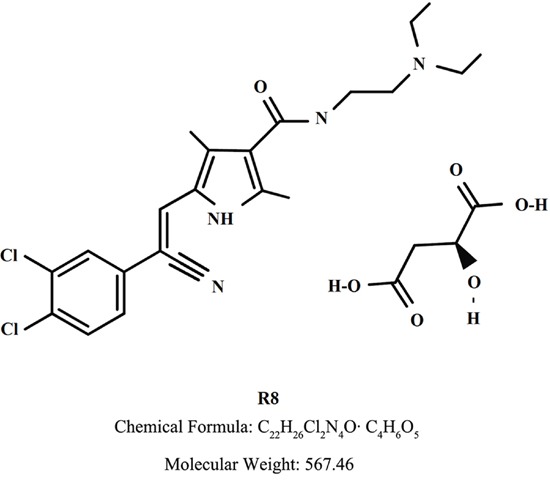
Chemical structure of R8

In the present work, we evaluated the *in vitro* inhibitory effect of a novel multikinase inhibitor, namely R8 (Figure 1), on several different types cancer cells. We further focused on the mechanism for its inhibition on lung cancer cells specifically, which showed distinct characteristics compared to Sunitinib, a clinically available MKI which has been investigated extensively in many types of cancer including lung cancer, either used alone or in combination with other therapeutics [9–11].

## RESULTS

### R8 significantly inhibited proliferation of non-small lung cancer cell line in a dose-dependent manner

**Figure 2:**
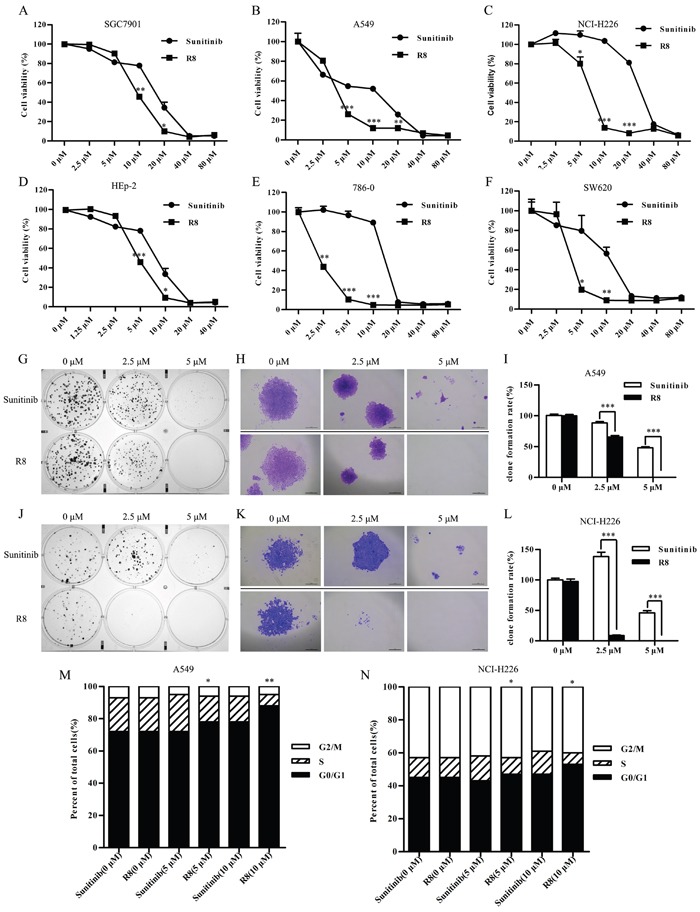
R8 significantly inhibited cell proliferation and colony formation than Sunitinib **(A, F)** Cell viability was detected by CCK-8 assay after six cell lines treated with Sunitinib or R8 for 48 h. (A) SGC7901, **(B)** A549, **(C)** NCI-H226, **(D)** HEp-2, **(E)** 786-0, (F) SW620. **(G, J)** R8 treatment of A549 (G) and NCI-H226 (J) significantly decreased numbers of colonies than Sunitinib after 14 days culture at concentration of 2.5 μM and 5 μM. **(H, K)** Typical colony morphology of A549 (H) and NCI-H226 (K) generated from single cell. Scale bar=20 μm. **(I, L)** Colony formation rate analysis of A549 (I) and NCI-H226 (L) treated with Sunitinib or R8. **(M, N)** Higher G0/G1 phase cell cycle arrest was induced after A549 and NCI-H226 cell lines treated with R8 for 24 h than Sunitinb. Data were presented as means ± SD of three independent experiments (^*^ *P*<0.05, ^**^ *P*<0.01, ^***^ *P*<0.001).

In order to evaluate efficacy of R8 on inhibiting cell proliferation, we selected clinically available MKI Sunitinib as a control, which was reported to be successful in inhibiting proliferation of variety kinds of tumors. We selected six cell lines, list as follows: human gastric carcinoma cells SGC7901, non-small lung cancer cell line A549 and NCI-H226, laryngeal carcinoma cell line HEp-2, renal adenocarcinoma cell line 786-0, colorectal cancer cell line SW620. As indicated by the cell viability curves (Figure 2A–Figure 2F), both Sunitinib and R8 inhibited proliferation of six cell lines in a dose-dependent manner. Surprisingly, R8 showed lower IC_50_ values than Sunitinib. The IC_50_ values of R8 were 9.61, 3.80, 6.78, 4.88, 2.25 and 4.05 μM, whereas IC_50_ values of Sunitinib were 15.01, 7.04, 28.11, 7.58, 13.75 and 10.14 μM in SGC7901, A549, NCI-H226, HEp-2, 786-0 and SW620 cell lines, respectively.

Furthermore, the long-term effect of R8 on cell survival was determined by colony formation assay. The results further confirmed that R8 was more significantly in inhibiting the proliferation of both A549 (Figure 2G–Figure 2I) and NCI-H226 (Figure 2J–Figure 2L) than Sunitinb.

### R8 induced G0/G1-phase cell cycle arrest and inhibited cell migration in non-small lung cancer cells

**Figure 3:**
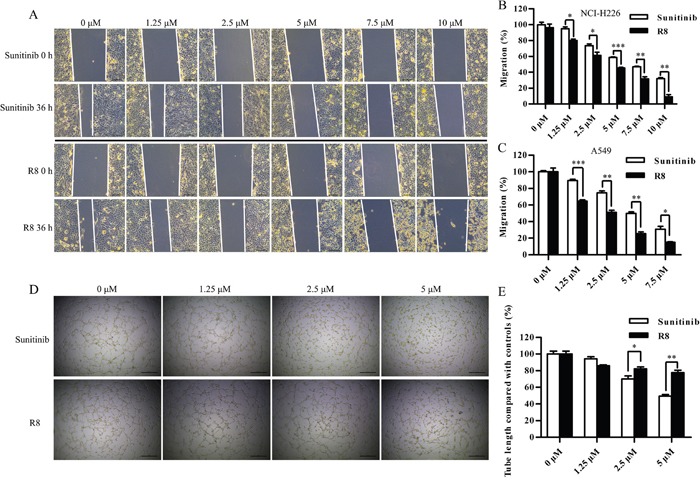
R8 inhibited cell migration of A549 and NCI-H226, but was less efficient in inhibiting tube formation **(A)** Micrographs showed the partial closing gap of NCI-H226 after treated with various concentrations of R8 or Sunitinib. Scale bar=10 μm. **(B, C)** Statistic analysis of cell migration after NCI-H226 and A549 treated with different doses of R8 or Sunitinib for 36 h. **(D, E)** Tube formation assay of HUVECs treated with different doses of Sunitinib or R8. Data were presented as means ± SD of three independent experiments (^*^ *P*<0.05, ^**^ *P*<0.01, ^***^ *P*<0.001).

To identify the mechanism underlying R8-mediated inhibition of proliferation, cell cycle analysis was performed. R8 caused enhanced accumulation of cells in G1 phase and decrease in S phase than Sunitinib in A549 (Figure 2M) and NCI-H226 (Figure 2N). Significantly induction of G0/G1 phase arrest may be one of reasons to answer why R8 was more competent in inhibiting cell proliferation. Since migration and angiogenesis are essential for tumor metastasis, so next we investigated whether R8 could inhibit cell migration ability. As shown in Figure 3, R8 significantly inhibited NCI-H226 (Figure 3A, Figure 3B) and A549 (Figure 3C) migration in a dose-dependent manner than Sunitinib. But when we evaluated tube formation of HUVECs treated with Sunitinib or R8, R8 was less efficient in inhibiting VEGF pathway than Sunitinib (Figure 3D, Figure 3E).

### R8 inhibited growth factor stimulated receptor phosphorylation

**Figure 4:**
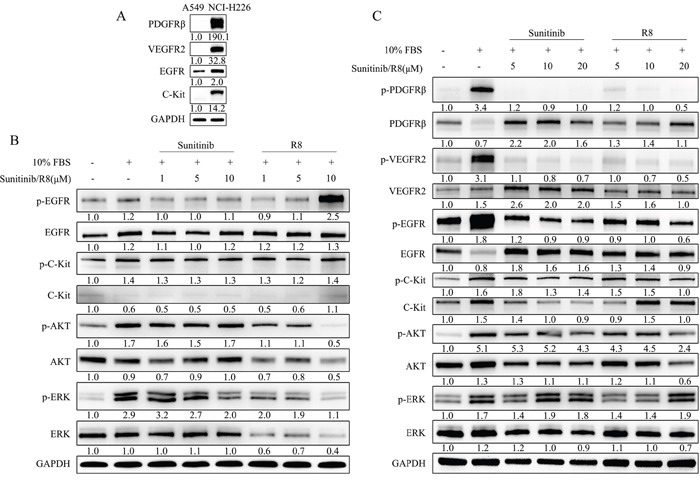
R8 significantly inhibited phosphorylation of receptor tyrosine kinases and down-stream AKT than Sunitinib in a dose-dependent manner **(A)** Comparison of expression level of PDGFRβ, VEGFR2, EGFR, C-Kit between A549 and NCI-H226 cell lines. All samples were 30 μg/well, except for EGFR (20 μg/well). **(B)** A549 cell line was incubated with various concentration of R8 or Sunitinib for 1 h, western blot analyzed phosphorylated EGFR, C-Kit, AKT, ERK after stimulation. **(C)** NCI-H226 cell line was incubated with various concentration of R8 and Sunitinib for 1 h, western blot showed phosphorylated PDGFRβ, VEGFR2, EGFR, C-Kit, AKT, ERK after stimulation. The protein densitometry values showed in the figure were normalized to their respective house-keeping protein GAPDH.

Above data indicated R8 could inhibit A549 and NCI-H226 cells proliferation and migration, but the IC_50_ values in two cell lines were different, NCI-H226 cell line seemed more resistant to drug treatment. So next we compared the expression levels of PDGFRβ, VEGFR2, EGFR, C-kit between A549 and NCI-H226. Undoubtedly, NCI-H226 expressed high level of totally four receptors, especially for EGFR and PDGFRβ, whereas A549 only with medium level of EGFR and low level of C-Kit expression (Figure 4A).

So next, we evaluated the phosphorylated status of tyrosine kinase receptors in A549 and NCI-H226 after exposed to Sunitinib or R8 for 1 h, respectively. Sunitinib treatment showed slight decrease in p-EGFR but no change with p-C-Kit, whereas R8 (10 μM) treated A549 caused an up-regulation in p-EGFR. P-AKT and p-ERK were activated after stimulation of 10% FBS, and Sunitinib inhibited phosphorylation of p-ERK in a dose-dependent manner whereas no change with p-AKT. Although R8 caused up-regulation of p-EGFR, dose-dependent inhibition of p-AKT, total AKT, p-ERK, total ERK were observed in R8 treated A549 cell line (Figure 4B).

For NCI-H226, which expressed abundant receptors, 10% FBS stimulation for 10 min caused significant up-regulation of four receptors. Sunitinib and R8 inhibited p-PDGFRβ, p-VEGFR2, p-EGFR, p-C-Kit expression in a dose-dependent manner. Although Sunitinib and R8 down-regulated p-EGFR and p-C-Kit, but was not so effective than p-PDGFRβ, p-VEGFR2.

P-Akt and total AKT were decreased in R8 (20 μM) treated NCI-H226 cells, but only a slight decrease of p-AKT in Sunitinib (20 μM) treated groups (Figure 4C).

### R8 induced autophagy and apoptosis

Since treated with Sunitinib and R8 caused a decline of phosphorylated receptors which may induce cell starvation, accompanied with down-regulated p-AKT, both of which may result in autophagy.

**Figure 5:**
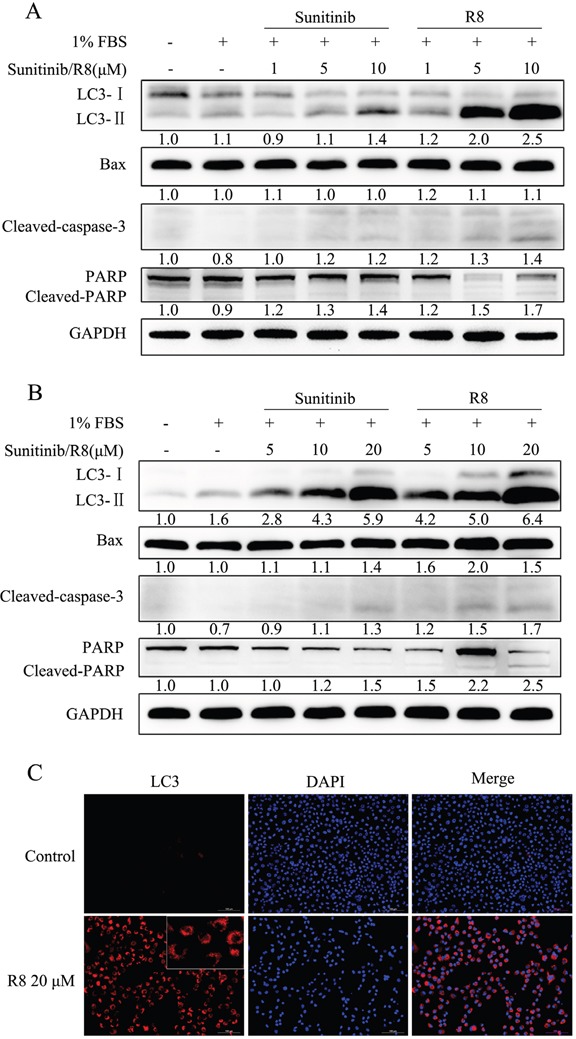
Strong autophagy was induced after A549 and NCI-H226 treated with high concentration of R8 for 48 h, as well as apoptosis **(A)** A549 cell line treated with different doses of Sunitinib or R8 for 48 h, western blotting analysis of the level of Bax, LC3, cleaved- caspase-3, PARP. GAPDH was used as a loading control. Experiments were repeated for three times and the data showed the representative results. **(B)** Significant activation of apoptosis and autophagy of NCI-H226 cell line was observed via western blotting of Bax, cleaved- caspase-3, PARP and LC3-II after treated with different doses of R8 for 48 h, especially for the high concentration group. **(C)** Immunofluorescence of LC3 for NCI-H226 cell line treated with R8 for 48 h. Red-punctate fluorescence of LC3-II could be detected in the R8 20 μM treated group.

So next we analyzed autophagy and apoptosis related proteins by Western blotting. LC3-II was a good indicator of autophagosome formation. For A549, R8 (5 μM, 10 μM) induced significant increase of LC3-II than Sunitinib in a dose-dependent manner. Apoptosis related proteins, such as cleaved-caspase-3 and cleaved PARP, were observed after treated with Sunitinib or R8 for 48 h, but no significant up-regulation of Bax, a pro-apoptotic molecule (Figure 5A). For NCI-H226, the expression of LC3-II was up-regulated in a dose-dependent manner both in sunitinb and R8 treated group, whereas R8 showed more potent in accumulating of LC3-II. Significant cleavage of caspase-3 and PARP, up-regulation of Bax were observed in R8 treated cells than Sunitinib (Figure 5B).

Since there were many vacuole-like bubbles in cytoplasm after NCI-H226 treated with R8 for 48 h compared with controls, so we performed LC3 immunofluorescence to distinguish cytosolic LC3-I and autophagosome-associated LC3-II. For NCI-H226 exposed to 20 μM R8, red-punctate LC3-II was observed in the cytoplasm, which indicated the formation of autophagosome (Figure 5C).

### R8 induced type II programmed cell death

In both A549 and NCI-H226 cell lines, R8 induced significant autophagy and apoptosis than Sunitinib. But the role of autophagy induced by Sunitinib or R8 was not clear. So we first incubated cells with autophagy inhibitor 3-Methyladenine (3-MA) or chloroquine phosphate (CQ) before drug treatment.

**Figure 6:**
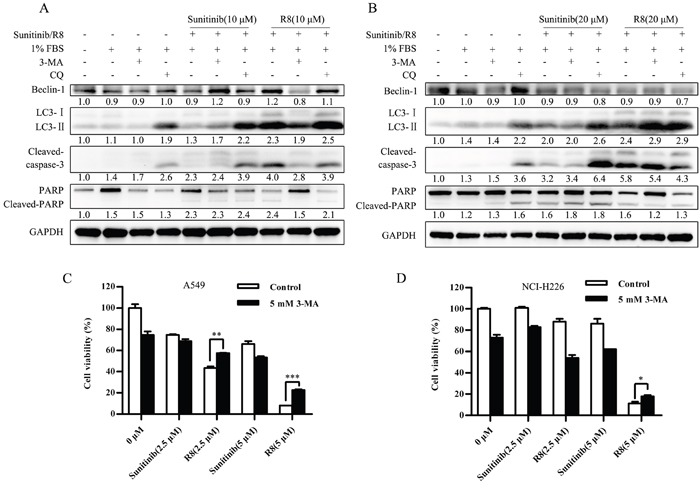
Self –protected role of Sunitinib induced autophagy whereas cell death mechanism of R8 induced autophagy **(A, B)** Autophagy inhibitor 3-methyladenine (3-MA) and chloroquine (CQ) were used to evaluate the role of autophagy induced by Sunitinib and R8, respectively. Western blotting analysis of the level of Beclin-1, LC3, cleaved-caspase-3, PARP in A549 (A) and NCI-H226 (B) cell line. Experiments were repeated for three times and the data showed the representative results. **(C, D)** Cell viability was detected by CCK-8 assay after A549 (C) and NCI-H226 (D) cell lines treated with combination of Sunitinib or R8 and autophagy inhibitor 3-MA (5 mM) for 48 h.

For A549, inhibition of autophagy by 3-MA or CQ resulted in increasing of cleaved caspase-3 and PARP, which indicated autophagy induced by Sunitinib in A549 was a self-protected autophagy. Once inhibited autophagy, apoptosis would increase. On the other hand, for R8 treated cells, 3-MA pre-treatment resulted in decreasing of Beclin-1, LC3-II, cleaved caspase-3 and PARP, which indicated that the autophagy induced by R8 was death-leading (Figure 6A). Once inhibited autophagy, cell would survive. CQ pre-treated A549 only showed slight function in inhibiting cell-death autophagy, which indicated class III-PI3K was more important in R8-induced autophagy.

For NCI-H226, 3-MA or CQ treatment also enhanced Sunitinib induced apoptosis, marked by up-regulation of cleaved caspase-3 and PARP. In R8 treated cells, 3-MA treatment only decreased 19 KD cleaved caspase-3 and PARP without down-regulation of LC3-II. Whereas CQ treatment caused significant down-regulation of Beclin-1, cleaved caspase-3 and PARP (Figure 6B). So in NCI-H226, CQ was more potent in inhibiting R8 induced autophagy, which indicated fusion with lysosome was more important in R8-induced autophagy of NCI-H226 cell line.

Besides, cell viability was up-regulated after A549 (Figure 6C) and NCI-H226 (Figure 6D) treated with combination of R8 and 3-MA, but not for the Sunitinib treated cells.

In conclusion, Sunitinib induced a self-protected autophagy, protected cells from apoptosis; whereas R8 induced autophagic cell death, which contributed to apoptosis.

## DISCUSSION

In this study, we observed the inhibition of a novel MKI R8 on cancer cells *in vitro*, with comparison to Sunitinib, one of the widely used MKIs in clinic, and explored the possible mechanism by which R8 took effect. As a class III/V multikinase inhibitor, Sunitinib was reported to suppress various kinds of tumors, such as human metastatic renal cell carcinoma, lung carcinoma, glioblastoma, melanoma [12–15] by inhibiting the activation of VEGFR-1, 2 and 3, PDGFR-α, β, Kit, Flt3 and RET. It has been approved by FDA to treat advanced renal cell cancer and imatinib mesylate-intolerant or resistant gastrointestinal stromal tumor since 2006 [16, 17].

Our results showed that R8 is more potent than Sunitinib in suppression of all types of cancer cells tested in this study, with much lower IC_50_ values when compared to Sunitinib. Moreover, when specifically focused on lung cancer, one type of cancer with high morbidity and mortality, R8 exhibits stronger inhibition than Sunitinib on colony formation, cell cycle arrest and migration of lung cancer cells A549 and NCI-H226, although less effective than Sunitinib in inhibiting tube formation of endothelial cells. PI3K-Akt-mTOR has been identified as a key signal pathway in regulating various aspects such as cancer cell proliferation, migration, apoptosis and autophagy [18, 19], and in this study we did observed that R8 induced significant down-regulation of p-AKT and total-Akt than Sunitinib did. Because of its key role in promoting cell cycle from G1 to S phase, down-regulated p-AKT resulted in cell cycle arrest at G0/G1 phase and proliferation inhibition.

Suppression of the Akt/mTOR pathway may also trigger autophagy and apoptosis [20–22]. Autophagy represents an important cell physiologic process in response to starvation, hypoxia, ER stress and oxidative stress. Autophagosomes engulf cellular organelles, such as mitochondria, ribosome, then fuse with lysosome to recycle energy used for cell survival during stress [23]. Generally, autophagy is considered as a way to protect cells from apoptosis [24–26]. Nonetheless, if stress over a safe threshold, autophagy can lead to cell death, called autophagic cell death or type II programmed cell death [27–29]. Indeed, several studies have confirmed the complex relationship between apoptosis and autophagy during the progress of cell treated with chemotherapeutic agents [30–33]. In this study we checked apoptosis and autophagy status of cancer cells treated with Sunitinib or R8 after 48 h. Because of down-regulation of PI3K-Akt-mTOR pathway, R8 induced significantly autophagy than Sunitinib in a dose-dependent manner marked by accumulation of LC3-II. Bcl-2 family proteins were activated to trigger apoptosis, resulted in up-regulation of Bax, cleavage of caspase-3 and PARP [34]. In the current study, we observed that suppression of R8-induced autophagy resulted in attenuated cleavage of caspase-3 and PARP, while the cleavage of caspase-3 and PARP were elevated on inhibition of Sunitinib-induced autophagy. The different sensitivities to 3-MA and CQ observed in this study were due to different mechanisms the two inhibitors involved in autophagy. Although both 3-MA and CQ are autophagy inhibitors, they block different steps of autophagy. 3-MA suppresses an early stage of autophagy by inhibiting the class III PI3K to block the production of PI3P, which is essential for early autophagosome formation [35]. CQ is a lysosomotropic agent which blocks the late stage of autophagy by elevating the lysosomal pH, inhibiting autophagosome fusion with lysosome and autophagosome degradation [36]. Taken together, these results suggested that Sunitinib-induced a pro-survival autophagy to evade cytotoxicity in lung cancer cells, similar to that in ovarian cancer [37], whereas R8-induced autophagy may have synergetic effect on cell death with apoptosis.

Our results also showed some differences between A549 and NCI-H226. NCI-H226, with more abundant receptor tyrosine kinases, showed more resistant to TKIs with higher IC_50_ than A549, while A549, with less hyper-activated tyrosine kinase receptors, was more sensitive to R8. Interestingly, we found that the inhibition of EGFR phosphorylation in A549 was not only independent of the dose of R8, but even showed up-regulation of p-EGFR when treated with higher dose of R8. Whether this is just a feedback of self-protective response to the severe stress or by some other specific mechanisms need to be investigated in the future. Furthermore, the differences between the two cell lines on critical steps during autophagy may also be due to the different responses to RTK inhibition. For A549, EGFR is a prominent tyrosine kinase. Activated EGFR phosphorylates Beclin-1 at three different tyrosine residues, Y229, Y233 and Y352, provokes the association of Beclin-1 with Rubicon/Bcl-2, which results in reduced autophagy. Exposure of A549 to tyrosine kinase inhibitors restores the Beclin-1-VPS34 interaction and triggers an increase in autophagy, but it is independent of mTOR, modulation of the Beclin-1-Rubicon/Bcl-2 complex is dominant in the EGFR-mediated autophagy. In NCI-H226 cell line, PDGFR can not induce Beclin-1 phosphorylation, but can strongly activate autophagy by the PI3K-mTOR pathway [38–40]. Thus, the mechanism of autophagy regulation, by EGFR-Beclin-1 or PI3K-mTOR, may be an important factor to decide critical steps in cell fate.

Notably, we demonstrated in this work that R8 can significantly inhibit the phosphorylation of PDGFRβ and VEGFR2. Previous studies have shown the association between dysregulation of these two RTKs and chemoresistance: increased PDGFRβ and VEGFR2 protein levels were associated with resistance to platinum-based chemotherapy and adverse outcome of ovarian cancer patients [41]; increased VEGFR2 gene copy was associated with chemo-resistance and shorter survival in non-small cell lung cancer patients who received platinum adjuvant chemotherapy after surgical resection [42]. Moreover, pretreatment with VEGFR and PDGFRβ inhibitors can inhibit tumor growth in a mouse colorectal carcinomatosis model [43]. Therefore, malignancies with dysregulated PDGFRβ and VEGFR2 may benefit from R8 treatment due to the potent inhibition on these RTKs.

In conclusion, current work showed that the novel compound R8 is a potent multikinase inhibitor which can effectively inhibit the phosphorylation of PDGFRβ, VEGFR2, EGFR and C-Kit. R8 shows more *in vitro* suppression on lung cancer cells than Sunitinib does by the synergetic effect of autophagic cell death and apoptosis. This provides the potential that R8 alone may serve as a good chemotherapeutic drug without co-administration of an autophagy inhibition drug to enhance the therapeutic efficacy which is suggested for many chemotherapeutic drugs. Further *in vivo* assays such as non-small cell lung cancer patient-derived xenograft models which express high level of PDGFRβ, VEGFR2, EGFR, C-Kit will be carried out to demonstrate that whether R8 can be developed into a new member of MKI drug family.

## MATERIALS AND METHODS

### Chemicals and reagents

Sunitinib (PubChem CID: 6456015, purity: 99.64%) was from Nanjing First Pharmaceutical Co., Ltd (Nanjing, China), R8 was synthesized by WuXi AppTec (Shanghai, China). 3-Methyladenine (3-MA, PubChem CID: 1673) was purchased from Selleck Chemicals (Houston, Texas, USA), Chloroquine Phosphate (CQ, PubChem CID: 64927) was purchased from Sigma (St. Louis, MO, USA).

Cell counting kit-8 was obtained from Dojindo (Tokyo, Japan). PI/RNase Staining Buffer was obtained from BD Bioscience (San Diego, CA, USA). Matrigel® Basement Membrane Matrix was obtained from Corning (Corning, NY, USA). Dc Protein assay kit and TGX Stain-free FastCast acrylamide Kit was purchased from Bio-Rad (Hercules, CA, USA). Complete^™^ Protease Inhibitor Cocktail Tablet and Phosphatase Inhibitor Cocktail 2 were purchased from Santa Cruze (Santa Cruz, CA, USA) and Sigma (St. Louis, MO, USA), respectively. BSA was purchased from Amresco (Solon, OH, USA). Immobilon^™^ western chemiluminescent HRP substrate was purchased from Millipore (Billerica, MA, USA). Antibodies for Phospho-PDGF Receptor β (Tyr751), PDGF Receptor β, Phospho-VEGF Receptor 2 (Tyr1175), VEGF Receptor 2, Phospho-C-Kit (Tyr719), C-Kit, Phospho-EGF Receptor (Tyr1068), Phospho-Akt (Ser473), Akt, Phospho-p44/42 MAPK (Erk1/2) (Thr202/Tyr204), p44/42 MAP Kinase, Bax, PARP, LC3 and Beclin-1 were all purchased from Cell signaling technology (Beverly, MA, USA). Antibody against EGFR was purchased from Abcam (Cambridge, MA, USA). HRP-conjugated Monoclonal Mouse Anti-GAPDH was purchased from KangChen Biotech (Shanghai, China). Secondary antibodies were purchased from Santa Cruz Biotechnology (Santa Cruz, CA, USA).

### Cell culture

Human gastric carcinoma cells SGC7901, non-small cell lung cancer cell line A549 and NCI-H226, laryngeal carcinoma cell line HEp-2, renal adenocarcinoma cell line 786-0, colorectal cancer cell line SW620 were obtained from American Type Culture Collection (Manassas, VA, USA). Cells were maintained in RPMI-1640 medium, supplemented with 10% fetal bovine serum (FBS) at 37°C in a humidified incubator with 5% CO_2_, Primary human umbilical vein endothelial cell line (HUVEC) was a kind gift from Professor Jianan Wang (Zhejiang University School of Medicine, China).

### Cell viability assay

R8 and Sunitinib were dissolved in ddH_2_O or RPMI-1640 medium respectively, to give a storage concentration of 20 mM and stored at −20°C for no more than one week. For A549, cells were seeded at a density of 8×10^3^ per well in 96-well plate and cultured for 24 h. After 24 h, 10% FBS-RPMI1640 was removed, cells were starved overnight. Then, cells were exposed to different concentration of R8 or Sunitinib (range from 2.5 μM-80 μM) for 1 h before stimulation. Subsequently, cells were stimulated with same volume of 1% FBS-RPMI1640 and cultured in the presence of R8 or Sunitinib. After 48 h, cell viability was assessed by cell counting kit-8 assay as described previously [44]. The IC_50_of R8 and Sunitinib were calculated based on the cell viability curve.

### Cell cycle assay

A549 (4×10^5^/well) and NCI-H226 (5×10^5^/well) cells were seeded in 6-well plate and cultured for 24 h. All procedures were the same as cell viability assay except for that we harvested the cells at 24 h after stimulation. Then cells were fixed with 75% ethanol overnight followed by RNase treatment and propidium iodide (PI) staining as the instruction of PI/RNase Staining Buffer indicated. Cells were subjected to flow cytometer (FACSCalibur, BD, San Jose, CA, USA) and analyzed using CELL Quest 3.0 software (BD, NJ, USA).

### Colony formation assay

A549 (500/well) and NCI-H226 (800/well) cells were seeded in 6-well plate and cultured for 24 h. After serum-starved overnight, cells were exposed to different concentrations of R8 and Sunitinib for 1 h then stimulated with same volume of 10% FBS-RPMI1640. After that, cells were cultured for 14 days to allow colony formation. Colonies were fixed with 4% PFA for 15 min then stained with 1% crystal violet for 15 min. Colony consisting of 100 or more cells was scored [45]. The results were photographed by ChemiDoc XRS+ image system of Bio-Rad (Hercules, CA, USA) and Lecia DMIL LED inverted microscope (Wetzlar, Germany).

### Migration assay

A549 (6×10^5^/well) and NCI-H226 (5×10^5^/well) were seeded in 6-well plate and cultured for 24 h. After that medium was replaced by RPMI-1640 medium without FBS, cells were starved for 24 h. Then on the third day, 10 μl pipette tip was used to scribe gaps in each well. Scratched cells were washed with PBS for three times. Simultaneously, the gaps were photographed, set as 0 h. Finally, different concentrations of R8 and Sunitinib were added into the medium. Photographs were captured at 12, 24, 36 h by inverted microscope for the same position [46]. The migrated distances were analyzed by Image-pro plus software.

### Tube formation assay

Matrigel basement membrane matrix was placed in 4°C overnight before use. On the following day, a 96-well plate was coated with 50 μl Matrigel on ice and incubated at 37°C for 30 min. Then HUVECs (10^4^/well) were added in serum-free medium in the absence or presence of various concentrations of R8 or Sunitinib. Each conditional group contained 5 wells. Pictures of representative ×10 fields were taken from 4 to 6 h. The length of endothelial tubes was measured by Image-Pro Plus to assess the tube formation ability.

### Western blot analysis

Cells were seeded in 100 mm culture dishes at a density of 1.5×10^6^ per dish. To evaluate the function of R8 on inhibiting growth factor stimulated receptor phosphorylation, cells were exposed to R8 for 1 h then harvested immediately on ice after simulated with 10% FBS-RPMI1640 for 10 min. To evaluate autophagy and apoptosis, cells were exposed to R8 and Sunitinib for 1 h then stimulated with 1% FBS-RPMI1640. Cells were harvested after 48 h treatment. For autophagy inhibitor, cells were treated with 3-MA (10 mM) or CQ (20 μM) for 2 h before exposed to R8 and Sunitinib.

The cell pellets were lysed in RIPA lysis buffer containing 100× Phosphatase Inhibitor Cocktail 2 and 50× Complete^™^ Protease Inhibitor Cocktail for 1 h on ice. Cell lysates were centrifuged at 14,000×g for 15 min at 4°C then supernatant extracts were collected. The protein concentration was determined by Dc Protein assay kit of Bio-Rad. Equal amount of proteins (30 μg/well) were separated by electrophoresis on 10–12% SDS-PAGE and transferred to PVDF membranes from Bio-Rad (Hercules, CA, USA). Membranes were blocked with 10% BSA-TBST for 1 h at room temperature and then incubated with primary antibodies at 4°C overnight. Next, membranes were incubated with an HRP-conjugated secondary antibody for 1 h at room temperature. The immune-reactive bands were visualized by the ECL kit from Millipore (Billerica, MA, USA).

### Statistical analysis

Each experiment was performed at least for three times, and the data were shown as means±standard deviation. The statistic difference was analyzed by two-tailed Student's *t*-test. *P* values <0.05 were considered to be statistically significant.

## Abbreviations

PDGFR: platelet-derived growth factor receptor
VEGFR: vascular endothelial growth factor receptor
EGFR: epidermal growth factor receptor
RTK: receptor tyrosine kinase
TKI: tyrosine kinase inhibitor
MKI: multi-target tyrosine kinase inhibitor
GIST: gastrointestinal stromal tumor
IC_50_: median inhibitory concentration
PCD: programmed cell death.
